# Sample Grating Distributed Feedback Quantum Cascade Laser Array

**DOI:** 10.1186/s11671-015-1115-9

**Published:** 2015-10-16

**Authors:** FL Yan, JC Zhang, CW Liu, N. Zhuo, FQ. Liu, SQ Zhai, ZG Wang

**Affiliations:** Key Laboratory of Semiconductor Materials Science, Institute of Semiconductors, Chinese Academy of Sciences, Beijing, 100083 China

**Keywords:** Sample grating, Quantum cascade laser array, Patterned AlN submount, 42.55.Px, 81.05Ea, 81.07.St

## Abstract

A sample grating distributed feedback quantum cascade laser array aim at broad tunability and enhanced side mode suppression ratios is presented. Utilizing a sample grating dependence on emission wavelength and epitaxial side down bonding technique, the array of laser ridges exhibited three separated single mode emissions centered at 4.760, 4.721, and 4.711 μm respectively, in continuous wave at room temperature. Side mode suppression ratios of >35 dB and continuous wave output powers of >10 mW per laser ridge were obtained.

## Background

Since it was firstly demonstrated in 1997 [1], distributed feedback quantum cascade laser (DFB-QCL) featuring narrow linewidth, high power, and simple fabrication process become extremely useful for spectroscopy [2, 3]. Very high continuous wave (CW) power and side mode suppression ratio (SMSR) DFB-QCL has also been demonstrated [4]. But a single DFB emitter has a very limited tuning range, usually achieved by changing the temperature of active core [5]. To achieve its wide tunability with single mode emission, QCL integrated with external cavity (EC) are demonstrated, but come at cost of bulky, vibration-sensitive, and high-quality optical components which makes it complex to build [6]. QCL integrated with dual-section cavities displays a wide tunability compared with conventional DFB-QCL, while the tuning range (~30 cm^−1^) is still limited for a monolithic device due to the vernier tuning nature [7, 8]. The DFB-QCL arrays monolithically integrating multiple DFB ridges have aroused extensively interest due to their wide tunability with single mode emission and gap-free tuning [9–11]. However, the performance of the array is still very limited, and it has not realized CW operation at room temperature. Actually, the present DFB-QCL array is confined to epitaxial side up bonding, thus, large amount of heat cannot be efficiently extracted from the devices due to the long thermal dissipation path between active cores and heat sink. Therefore, reducing the thermal dissipation path by epitaxial side down bonding technique is an efficient way to improve the heat extraction efficiency, which has been widely employed for a single laser. However, no results corresponding to epitaxial side down bonding of DFB-QCL array are reported up to now due to the complicated fabrication process of patterned submount.

In this paper, we fabricated a sample grating DFB array aim at broad tunability and enhanced SMSR. The array of laser ridges were designed to emit single mode emissions centered at 4.760, 4.721, and 4.711μm, respectively. By utilizing epitaxial down bonding technique with a patterned aluminum nitride (AlN, polycrystal, ceramics) submount, the laser ridges have realized CW operation at room temperature [12]. SMSR of >35 dB and CW output powers of 13 mW per DFB ridge were obtained.

## Methods

**Fig. 1:**
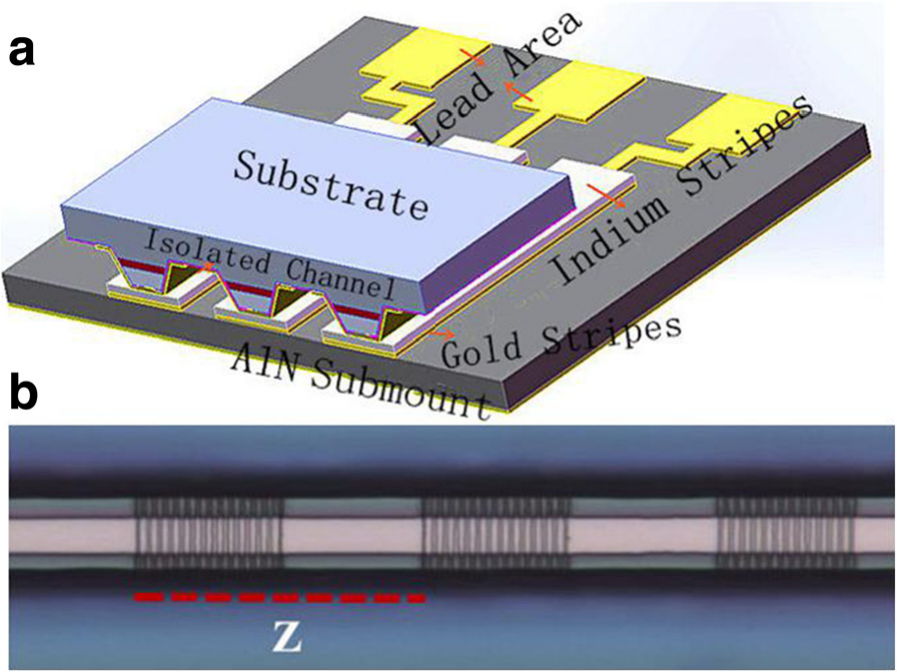
The schematics of **a** the DFB-QCL array is epitaxial side down bonded on a patterned AlN submount. **b** The optical microscope image of a sample grating DFB ridge, and *Z* is the sampled period

The QCL structure used in this letter, emitting at *λ* ~4.6 μm, is based on an In_0.669_Ga_0.331_As/In_0.362_Al_0.638_As so called double-phonon resonance design. The epitaxial growth and layer structure are identical to those given in ref. [13]. Device fabrication started with the definition of a sample DFB grating on upper InGaAs layer, as is similar to ref. [14]. The MBE-grown top cladding was first removed down to the upper InGaAs layer. Then, the based Bragg grating with a period of 0.701 μm and duty cycle (the ratio of grating peak to based grating period Λ) of 45 % was defined using holographic lithography technique. Three separate sampling periods (namely 9.9, 11.3, and 12.1 μm, respectively) with the same duty cycle (the ratio of grating area to sampling period) of 50 % were formed by the conventional optical photolithography and transferred by wet chemical etching to the depth of about 150 nm. The sampled grating of one DFB ridge is shown in Fig. 1b. Then, a 3-μm low-doped (Si, 2.2 × 10^16^ cm^−3^) InP layer, followed by a 0.15-μm gradually doped (Si, 1 × 10^17^ to 3 × 10^17^ cm^−3^) InP layer, and a 0.6-μm highly doped InP (Si, 5 × 10^18^ cm^−3^) cladding layer were accomplished in sequence as the upper cladding by metal organic vapor phase epitaxy (MOVPE) regrowth. Then, the DFB ridges, with a mean core width of 12 μm and spaced 170 μm apart, were processed using optical photolithography and nonselective wet chemical etching. The bottom and sidewalls of the DFB ridges were passivated with a 450-nm-thick SiO_2_ layer by chemical vapor deposition. Then, 40/250-nm-thick Ti/Au contact layers were evaporated by electron beam evaporation, followed by a 4-μm-thick electroplated gold layer. The 50-μm-wide electrical isolation trenches between adjacent DFB ridges were defined by wet etching of Au/Ti layers. After thinning the substrate down to about 120 μm, the backside of the wafer was deposited with Ge/Au/Ni/Au metals as substrate contact layer. The waveguide was then cleaved to 2-mm-long cavities, and the high reflectivity (HR) coating consisting of Al_2_O_3_/Ti/Au/Ti/Al_2_O_3_ (200/10/10/100 nm) was evaporated on the back facet. The entire laser size was about 1.6 mm × 2 mm.

To realize the epitaxial side down bonding of the array, a patterned submount was fabricated by photolithography and lift-off technique. The specified patterns were transferred on a 2″ round, 350-μm-thick AlN ceramic submount, and then, Ti/Au (40/150 nm) layers were evaporated. The gold stripes and lead area were left after removal of the residue metal by lift-off technique, followed by electroplating a 2-μm-thick gold layer to enhance the thermal spreading. The submount was finished by electroplating a 3-um-thick indium solder layer on the gold stripes. As shown in Fig. 1a, the DFB array was epitaxial side down bonded on the patterned submount with double-side alignment welding machine. The DFB ridges must be aligned with the indium solder stripes. And between them, there are isolated channels to separate the adjacent DFB ridges for their electrical isolation. Gold lead squares, electrical connecting with DFB ridges, were wire bonded on a custom-designed circuit board. And then, the array was mounted on a holder containing a thermistor to monitor and a thermoelectric cooler (TEC) to adjust the heat sink temperature. The output optical power from the uncoated facet of the array was measured with a calibrated thermopile detector placed directly in front of the laser facet. All measurements were taken under CW mode at room temperature.

## Results and Discussion

**Fig. 2:**
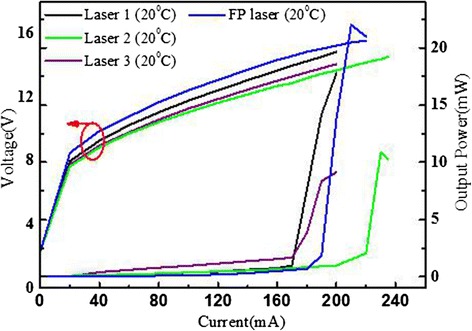
The power-current-voltage (*P*-*I*-*V*) curves of the DFB array with HR coated facets when operated in CW at 20 °C. The size of DFB ridge is 2-mm-long and 12-um-wide. The *blue* ones are *P*-*I*-*V* curves for a FP cavity laser from the same wafer with the same size when operated in CW mode at 20 °C

Figure 2 shows the power-current-voltage (*P*-*I*-*V*) curves of a 2-mm-long, 12-μm-wide, epitaxial side down bonded DFB ridges in the array at a temperature of 20 °C. The ridges, with the threshold current density of 0.708, 0.896, and 0.708 kA/cm^2^, emit up to the maximum power of 18, 12, and 9 mW, respectively. A Fabry-Perot (FP) cavity laser (the device size is the same as DFB arrays) shows a higher output power and slightly increased threshold current compared to the DFB devices. In addition, the small dynamic range and low threshold current density of the lasers may be caused by low doping level of active core since the device size was not very small [15, 16].

**Fig. 3:**
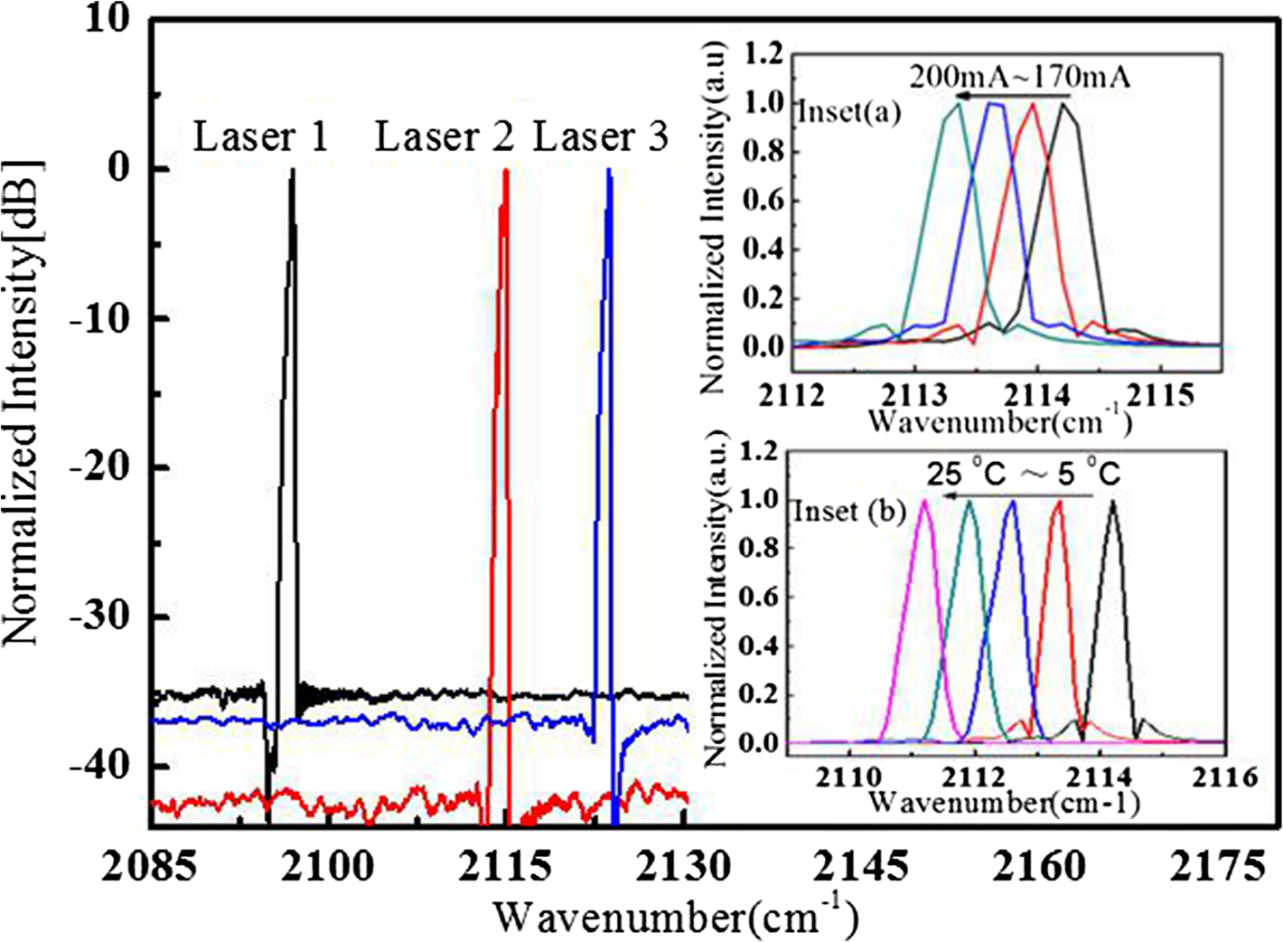
The spectrum of all three lasers in the array and the injected currents keep at 1.2 *I*_th_ in CW mode at 20 °C. *Inset (a)* and *inset (b)* the spectrum of no. 2 in the array with current changing from 170 to 200 mA at 20 °C and with the temperature changing from 5–25 °C at 200 mA, respectively

The emission spectrum of the array was measured using a Fourier transform infrared spectrometer (Nicolet 6700) with a resolution of 0.25 cm^−1^. Figure 3 shows all three DFB ridges in the array operating in single mode at the injected current of 1.2 *I*_th_ (threshold current). The measured wavelength of the DFB ridges in the array are 4.760 μm (designed at 4.800 μm), 4.721 μm (4.740 μm), and 4.711 μm (4.720 μm), which locate slightly left to the center of the designed +1st order Bragg modes. The reason is that we have overestimated the effective refractive index *n*_eff_ ~3.20, which is about 3.19 according to the measurement results. Such a high SMSR benefits from (i) the use of +1st Fourier spectrum sampled grating as mode selection mechanism, which is not affected by defect in the based Bragg grating region and (ii) the sample grating DFB lasers are much less sensitive to the facet phase effects than standard DFB lasers [7]. The temperature tuning coefficient of laser 2 is about 0.15 cm^−1^/K, which is comparable to conventional DFB lasers.

**Fig. 4:**
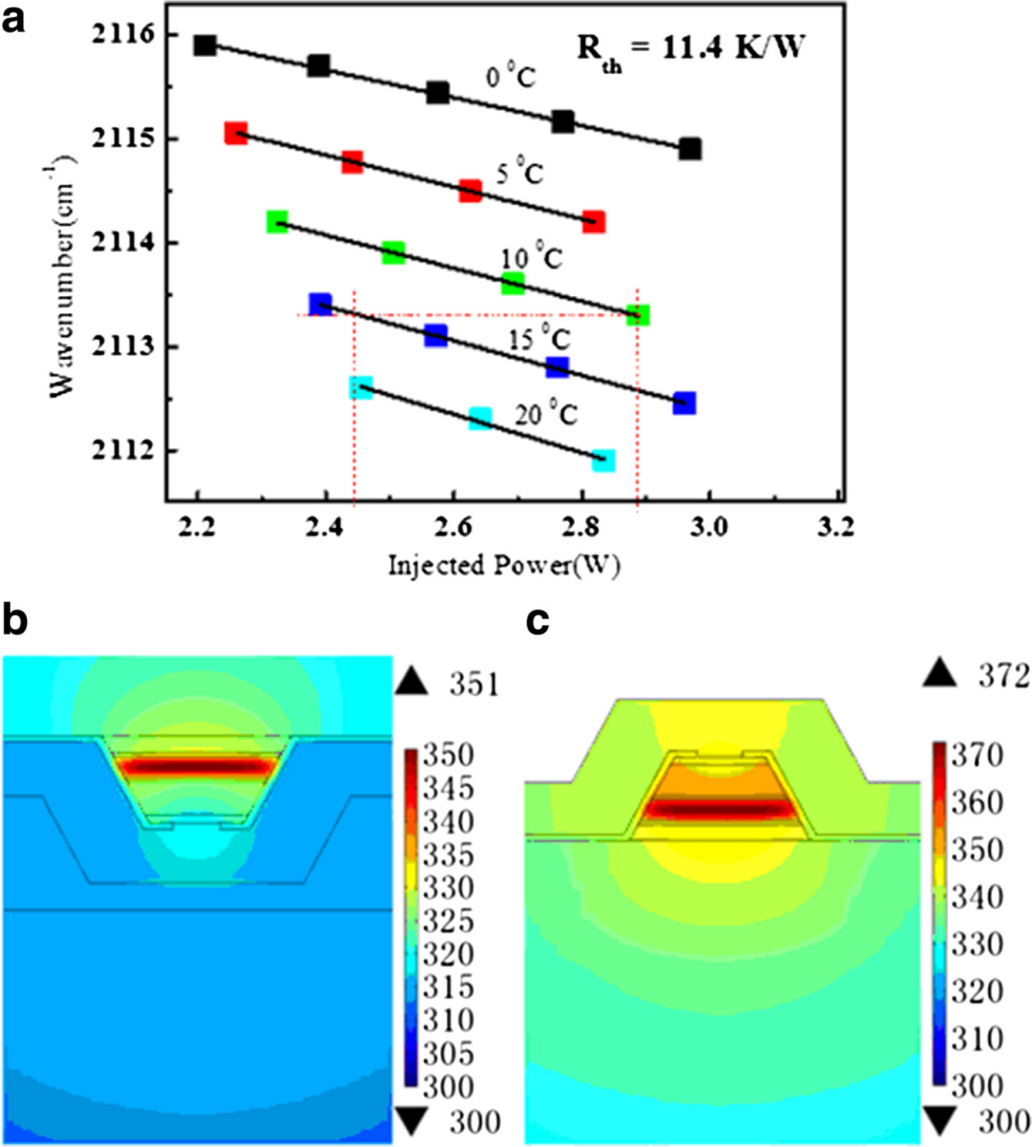
(**a**) The emission frequency of the no. 2 laser in the array as functions of the injected electrical power (*scattering dots*) and fitting results (*straight lines*) which give the parameter *R*_th_, including the thermal resistance and the tuning coefficient *β*. **b**, **c** Heat dissipation simulation results of epitaxial side down and up ridges. The injected powers keep at 4.25 W, and the heat sink temperatures keep at 300 K

The thermal resistance *R*_th_ of the device is deduced from the variation of the emission frequency as a function with the temperature of active region *T*_act_. The emission frequency changes as *υ* = *υ*_0_ + *βυТ*_act_, where *β* = (1/*υ*) (Δ*υ*/Δ*T*) is the tuning coefficient, *Т*_act_ = *T*_sink_ + *P*_elec_*R*_th_, where *T*_sink_ is the heat sink temperature and *P*_elec_ the injected electrical power [17]. As shown in Fig. 4a, fitting *υ* = *υ*_0_ + *βυT*_sink_ + *βυP*_elec_*R*_th_ using the experimental data of no. 2 laser in the array leads to the *R*_th2_ of 11.4 K/W and *β* ~−6.49e^−5^ K^−1^. Using the same method, *R*_th1_ and *R*_th3_ are fitting to be 12.5 and 11.4 K/W respectively, which are typical values for InGaAs/InAlAs QCL ridges without lateral regrowth [18].

For the presented epitaxial side down bonded DFB ridges, the maximum temperature to maintain CW operation is measured to be 25 °C. We simulated the heat dissipation of epitaxial side up and down bonded DFB ridges by a commercial finite element software (COMSOL). The maximum temperature difference between these two ridges is 21 °C as shown in Fig. 4b, c. The simulated *R*_th_ (~ΔT/P) for epitaxial side up and down bonded ridges are 16 and 11.3 K/W, respectively, which match very well with the previously reported values [19]. The temperature of active core can be expressed by *T*_core_ = *T*_sink_ + *R*_th_*P. For epitaxial side down ridges, the maximum temperature *T*_core_ (max) = 25 °C + 11.3 K/W*4.25 W = 73 °C. While for epitaxial side up ridges, the maximum temperature of heat sink is *T*_sink_ (max) = 73 °C − 16 K/W*4.25 W = 5.0 °C. Therefore, the epitaxial side up bonded DFB ridges can only be operated below 5.0 °C. To further decrease the thermal resistance of the device and improve the maximum operating temperature, we can change the polycrystal AlN into submounts of higher thermal conductivity (such as single crystal AlN, SiC, diamond) and use the buried laser ridges.

## Conclusions

In conclusion, we realized a DFB-QCL array utilizing sample grating and epitaxial side down bonding technique. The array of laser ridges exhibited three separated single mode emissions centered at 4.760, 4.721, and 4.711 μm respectively, operating in CW mode at room temperature. SMSR of >35 dB and CW output powers of > 10 mW per DFB ridge were obtained. The sample grating and patterned submount provide practical experience for the fabrication of high SMSR and wide tunable DFB array operating in CW mode at room temperature. Though it is still limited, the spectral coverage of the array can be extend by integrating more DFB ridges.

## Abbreviations

AlN: aluminum nitride
CW: continuous wave
DFB-QCL: distributed feedback quantum cascade laser
EC: external cavity
FP: Fabry-Perot
HR: high reflectivity
MBE: molecular beam epitaxy
*P*-*I*-*V*: power-current-voltage
SMSR: side mode suppression ratio
TEC: thermoelectric cooler
